# Image-Based Differentiation of Benign and Malignant Peripheral Nerve Sheath Tumors in Neurofibromatosis Type 1

**DOI:** 10.3389/fonc.2022.898971

**Published:** 2022-05-23

**Authors:** Jun Liu, Jing-Ning Huang, Ming-Han Wang, Zhen-Yang Ni, Wei-Hao Jiang, Manhon Chung, Cheng-Jiang Wei, Zhi-Chao Wang

**Affiliations:** ^1^ School of Medicine, Shanghai Jiao Tong University, Shanghai, China; ^2^ School of Electronic Information and Electrical Engineering, Shanghai Jiao Tong University, Shanghai, China; ^3^ Department of Plastic and Reconstructive Surgery, Shanghai Ninth People’s Hospital, School of Medicine, Shanghai Jiao Tong University, Shanghai, China

**Keywords:** neurofibromatosis type 1 (NF1), malignant peripheral nerve sheath tumors (MPNST), differential diagnosis, medical radiology image methods, future prospectives

## Abstract

Neurofibromatosis type 1 (NF1) is a dominant hereditary disease characterized by the mutation of the *NF1* gene, affecting 1/3000 individuals worldwide. Most NF1 patients are predisposed to benign peripheral nerve sheath tumors (PNSTs), including cutaneous neurofibromas (CNFs) and plexiform neurofibromas (PNFs). However, 5%-10% of PNFs will ultimately develop into malignant peripheral nerve sheath tumors (MPNSTs), which have a poor prognosis. Early and reliable differentiation of benign and malignant tumors in NF1 patients is of great necessity. Pathological evaluation is the “gold standard” for a definite diagnosis, but the invasive nature of the biopsy procedure restricts it from applying as a screening tool during the decades-long follow-up of these patients. Non-invasive image-based diagnostic methods such as CT and MRI are often considered essential screening tools for multiple types of tumors. For NF1 patients’ lifelong regular follow-ups, these radiological methods are currently used for tumor evaluation. However, no consensus was established on screening the malignant transformation of benign PNSTs. Moreover, novel technologies like radiogenomics and PET-MRI have not been well evaluated and fully adopted for NF1 patients. This review summarizes current studies of different imaging methods for differentiating benign and malignant tumors in NF1. Meanwhile, we discussed the prospects of the usage of new tools such as radiogenomics and PET-MRI to distinguish MPNST from benign PNSTs more precisely. Summarizing these findings will help clarify the directions of future studies in this area and ultimately contribute to the radiology images-based clinical screening of MPNST in NF1 patients and finally improve the overall survival rates of these patients.

## Introduction

Neurofibromatosis type 1 (NF1), a hereditary disorder that primarily affects the peripheral nervous system, has a prevalence of approximately 1:2500 to 1:3500 in individuals worldwide (1). NF1 is caused by the mutation of the *NF1* gene, and the classic clinical characteristics include café-au-lait macules, skinfold freckling, benign neurofibromas, brain tumors, iris hamartomas, and typical bony lesions (2). Among those symptoms, benign neurofibromas, including cutaneous neurofibroma (CNF) and plexiform neurofibroma (PNF), are among the most common features of NF1. Approximately 30%-50% of patients with NF1 have plexiform neurofibromas. As a benign tumor, disability and deformity are common for these patients due to the vast tumor volume. Moreover, 5%-10% of these PNFs have the capacity for transformation into malignant peripheral nerve sheath tumors (MPNSTs), which have a poor overall survival rate of typically less than 5 years (1). Early diagnosis of MPNST is essential for early treatment, which will ultimately improve the prognosis of the patients. Tissue biopsy is considered the definitive diagnostic method for these patients, but as an invasive method, it cannot serve as a screening tool to be applied throughout the lifelong follow-up of patients with NF1. There is an urgent need for non-invasive, widely-used, and economical tools for these patients.

Medical radiology methods such as computed tomography (CT), magnetic resonance imaging (MRI), and positron emission tomography-computed tomography (PET-CT) played significant roles in various types of tumors. Unlike biopsy, these image-based methods are noninvasive and more suitable screening tools. One of the main functions of medical imaging methods is to distinguish benign lesions from malignant tumors. A report suggested that ultrasound-based differentiation of malignant and benign thyroid nodules has promising potential for clinical use (3). Meanwhile, another study showed that Cone-beam CT was proposed as a novel approach to predict breast lesion malignancy (4). Actually, in NF1-PNSTs and NF1-related MPNSTs, these image-based methods are also considered essential and widely adopted in tumor diagnosis and evaluation. The above studies on other types of tumors suggest the potential of image-based methods serving as efficient, noninvasive, inexpensive, and widely available tools in the differentiation of benign PNF and MPNST (5). However, as NF1 tumors are relatively rare, clear indications of image-based distinction between benign and malignant NF1 have not yet been fully defined.

To identify current studies and possible future directions in this area, we conducted a systematic review of the literature on radiology image-based differentiation of benign and malignant tumors in NF1. This review comprehensively summarizes different image-based methods used in distinguishing benign from malignant NF1 tumors, including CT, MRI, PET-CT, and ultrasound. This review also discusses the combination of radiology images and multiple-omics disciplines, such as the potential of clinical usage of radiogenomics in this field. On this basis, we further discuss possible future directions of radiology image methods in NF1. Better clarification of these will contribute to the early differential diagnosis of MPNST from PNF and eventually improve the overall survival of these patients.

## Materials and Methods

### Search Strategy and Information Sources

This review was in line with recommendations from the Preferred Reporting Items for Systematic Reviews and Meta-Analyses (PRISMA) statement. The publications were identified by comprehensive searching of PubMed and our own reference library. Search terms included combinations of “Neurofibromatosis type 1,” “Malignant peripheral nerve sheath tumors,” “magnetic resonance imaging,” “Computed Tomography,” “PET imaging,” “ultrasound,” and “radiogenomics.”

### Study Selection, Data Collection, and Exclusion/Inclusion Criteria

Selection of material was limited to papers published in English. All of the publications were checked by at least two investigators. Patents, books and documents, case reports, and conferences were excluded. Also studies regarding only neurofibromatosis type 2 (NF2) or other unconcerned diseases were excluded. Studies related to differential diagnosis of benign and malignant tumors based on imaging characteristics, and correlation of genomics and radiology, are the inclusion criteria.

## Results

**Figure 1:**
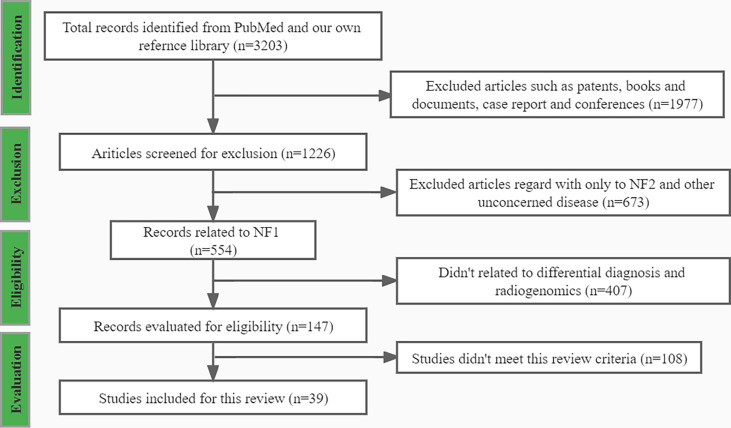
The flowchart of publications screening process.

Using the search strategies mentioned above, 3203 records were presented, and among which 39 records met this review criteria. After applying the exclusion criteria, 3165 publications were removed, including; (a) 1977 records, such as patents, books and documents, case reports, and conferences; (b) 672 records did not relate to NF1; (c) 407 records did not relate to differential diagnosis and radiogenomics; and (d) 108 records did not meet the inclusion criteria. A flowchart (**Figure 1**) demonstrates the screening process and study selection.

### Magnetic Resonance Imaging (MRI)

MRI is currently the preferred radiology image method for NF1. Compared to CT images, the MRI has a better resolution for this soft-tissue tumor. Based on the scarce clinical consensus, multiple current studies have already described the potential of MRI in the differential diagnosis of MPNST from NF1.

**Figure 2:**
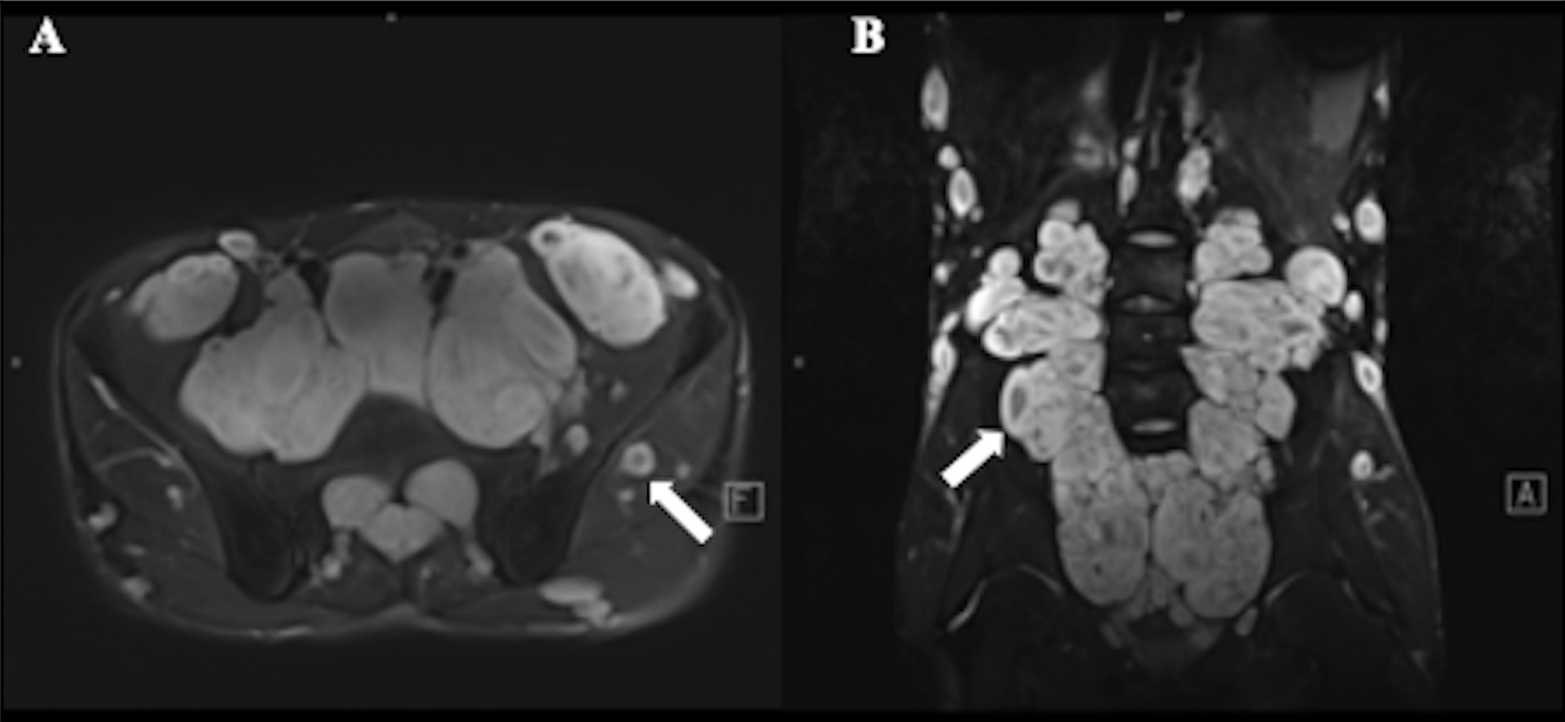
A 17-year-old male with plexiform neurofibroma (PNF) who presented multiple masses throughout the body. T2-weighted MR image showed a target sign (arrow) with peripheral area of high intensity and central area of low intensity. **(A)** Multiple nodules and lumps in the walking area of the bilateral femoral nerve, sciatic nerve, and obturator nerve; **(B)** Multiple nodules and lumps in the epidermis, subcutaneous and soft tissues of the abdomen and pelvis.

**Figure 3:**
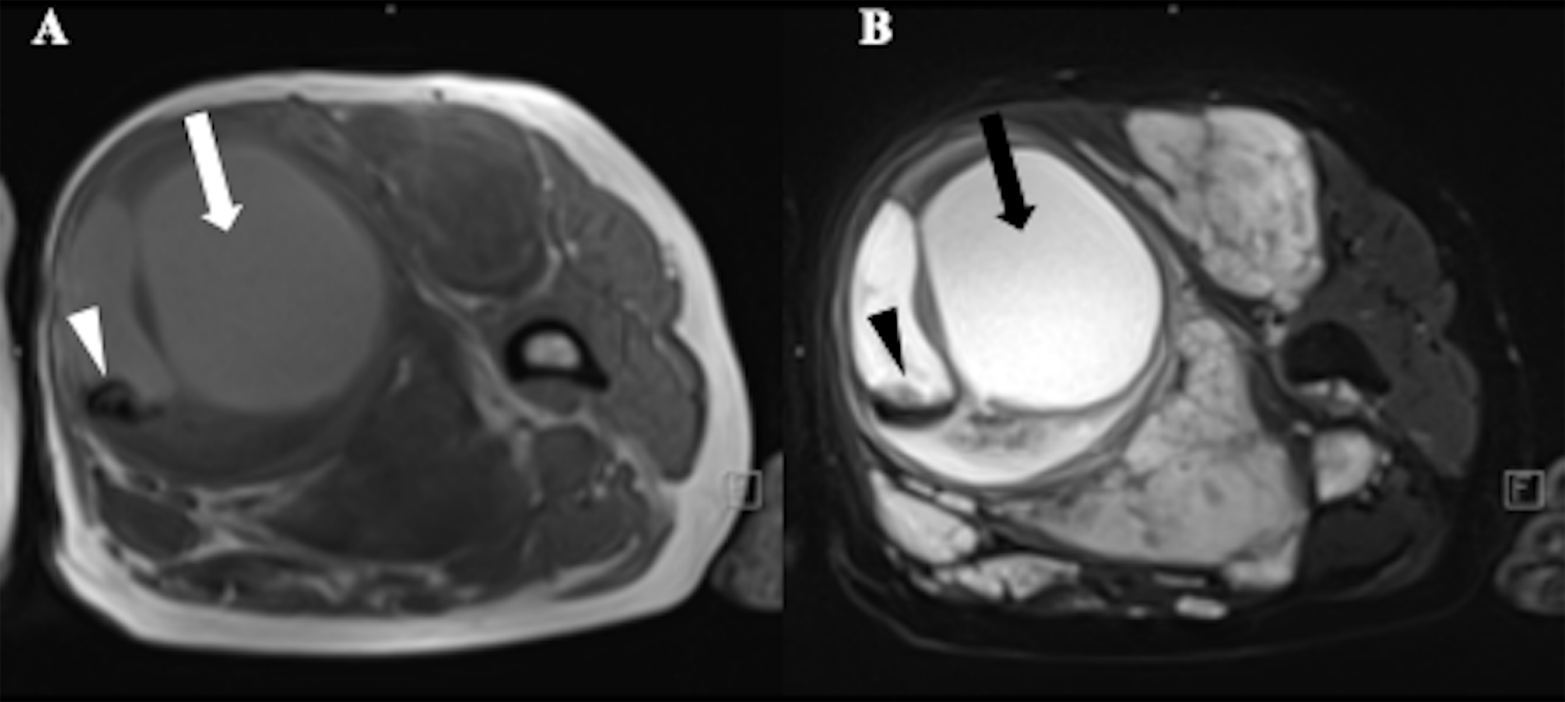
A 52-year-old female with Neurofibromatosis type 1 (NF1) who presented with a large mass in soft tissue of upper left thigh. MRI showed a heterogeneous signal with cystic change (arrows) and hemorrhage (arrowheads) in the mass, suggestive of malignant transformation. **(A)** T1-weighted MR image;**(B)** T2-weighted MR image.

Benign neurofibroma is a well-defined mass with high intensity on T2-weighted MRI images. A central area of low intensity (the “target sign”) in PNF lesions is sometimes observed, which is due to the presence of myxoid material peripherally and fibrous tissue centrally (**Figure 2**). Studies demonstrated that although not all benign tumors showed a “target sign,” it indicated the lesion as benign PNF once it occurred (6). Compared with PNFs, MPNSTs on T2 sequences were more extensive with an infiltrative margin. Moreover, the invasive growth of this malignant tumor resulted in a perifocal edema reaction which presented as “feathery” outside the tumor pseudo capsule (7). On T1-weighted images, it is hard to distinguish benign neurofibromas from MPNSTs due to the isointensity to adjacent muscles, but neurofibromas showed central focal enhancement and MPNSTs showed peripheral enhancement on T1-enhanced sequence after gadolinium (Gd) administration (7, 8). The underlying pathological mechanisms were that malignant transition of the tumor occurred with necrosis, hemorrhage, or both, leading to intratumoral cystic changes, accompanied by heterogeneity on MRI, but this rarely happens in neurofibroma (9) (**Figure 3**). In general, several key features mentioned above can be used to distinguish MPNST from benign neurofibroma, including the largest dimension of the mass, signal features of TI-weighted images and T2-weighted images, enhancement pattern, and cystic changes (**Table 1**). Junji Wasa et al. reported that the presence of two or more of the four features (the largest dimension of the mass, peripheral enhancement pattern, perilesional edema like zone, and intratumoral cystic lesion) had indicated malignant peripheral nerve sheath tumors with a sensitivity of 61% and a specificity of 90% (7). A meta-analysis of the included lesions involving at least 300 patients with NF1 (616 in total, some with NF1 features were not reported) showed that pooled and weighted sensitivity, specificity, and AUC values for MRI in detecting MPNSTs were 68%, 93%, and 0.89 when using feature combination, with specificity of perilesional edema and irregular being 94% and 90%, respectively (10).However, it is worth noting that morphological identification based on MRI is highly subjective. Furthermore, not all patients have the typical signs mentioned above on MRI images, and the scarcity of “atypical patients” also restricts the clinical usage and popularization of these features.

**Table 1:** Features of Neurofibroma and MPNST on MRI.

	Neurofibroma	MPNST
Lesion size	small	Large
Margin	well defined	Invasive or ill-defined
Signal features of T1-weighted images T2-weighted images	peripheral high with central low on the T2-WI (target sign)	peritumoral edema reaction show feathery outside the tumor a hyperintense signal on the T2-WI
	Heterogeneous on T1-WI	Heterogeneous on the T1-WI
Enhancement Pattern (Gd administration)	central enhancement on the T1WI	peritumoral edema presents edge enhancement on T2WI peripheral enhancement on the T1WI
cystic changes	few cystic changes	necrosis or hemorrhage
Whole-body MRI	/	more internal tumors
		larger volume
DW-MRI	/	lower diffusivity
		higher perfusion fraction

Beyond morphological features on MRI, functional MRI (fMRI), especially the diffusion-weighted image (DWI) based fMRI, has recently played an essential role in identifying MPNST as an auxiliary diagnostic technique. Well L et al. acquired axial respiratory-triggered echo-planar sequences with 11 diffusion gradient b-values and used DWI-derived parameters (e.g., ADC, IVIM) for diagnosis (6). They found that DWI exhibited better performance in the differentiation of benign and malignant peripheral nerve sheath tumors (MPNSTs) in patients with NF1 compared with only using morphological features determined by MRI (6). Ahlawat S et al. further found that the “target sign” was more frequently visible on high b-values DWI images and ADC images than on anatomic sequences (11). They thought that the absence of a “target sign” on DWI might further indicate a neurogenic neoplasm as a malignant lesion, but this study lacked histological confirmation in benign cases and failed to assess and explain the histological architecture of the “target sign” (11). The meta-analysis mentioned above reported ADCmin or ADCmean with or without feature combination had sensitivity of 88%, specificities of 94%, and AUC values of 0.97 (10). A further study has found that DWI/ADC mapping specificity is likely to be another valuable method for MPNST differential diagnosis (12).

Another essential point about NF1 is that patients with neurofibromas may have tumors all over the body, and whole-body tumor burden is another indicator for the risk of malignant transformation (13). Whole-body MRI is an efficient tool for the whole-body tumor burden evaluation. A study demonstrated its suitability as a tool for identifying concealed MPNST (14). Wenli Cai et al. used the dynamic-threshold (DT) level set three-dimensional segmentation method to perform whole-body MRI and calculated volume, breaking the shackles of traditional two-dimensional methods (15). This 3D method allowed us to analyze the number and volume of tumors, which can be more effective for reliably assessing the patients’ tumor burden (15). In addition, whole-body MRI can better track the occurrence and progression of tumors to assist doctors in better understanding the dynamic changes from PNF to MPNST (16). The regular surveillance by this method is especially essential for children because most PNF growth occurred at a young age, not in adulthood (17). By using whole-body MRI, various complications of tumors can be detected and treated before symptoms further developed and irreversible damage occurred (18).

### Computed Tomography (CT)

CT is an ideal examination method for observing bone, joint, and soft tissue lesions. Advanced computed tomography (CT) methods, such as CT perfusion and dual-energy CT, can help distinguish benign lesions from malignant head and neck tumors (19). However, there were no definitive CT diagnostic features reported that can be used to differentiate MPNST from benign PNF among NF1 patients until now (20). Nevertheless, our team developed a machine learning approach based on CT images that has recently shown great potential in differentiating MPNST from benign NF1 (20). This model, developed by combining machine learning technology with CT images, accurately distinguished malignancy-transformed lesions from benign neurofibromas of the head and neck. However, the limitation in training cohort hinders the accuracy of this model when applied to other parts of the body (20).

### PET Imaging

**Figure 4:**
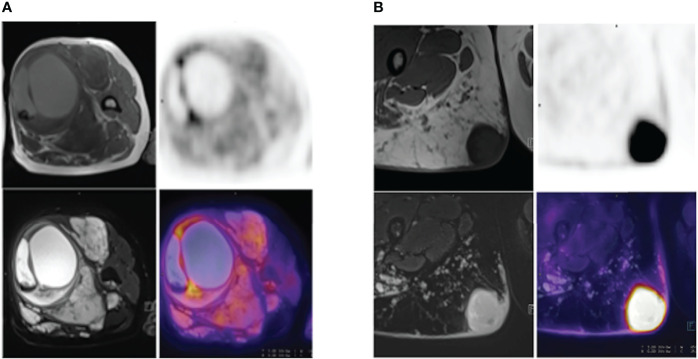
**(A)** A 52-year-old NF1 patient with benign PNF lesions, particularly in the left thigh. The lesion was located in the soft tissue of the upper segment of the left thigh, was approximately 8.7 cm-9.5 cm in size, with clear boundary, heterogeneous internal signal, visible cystic degeneration with hemorrhage, and increased FDG uptake at the septa, SUVmax=4.9. **(B)** A 28-year-old NF1 patient with a MPNST in the upper segment of the right thigh, measuring 3.9 cm – 3.8 cm, with well-defined borders and increased FDG uptake, SUVmax=14.4.

Positron emission tomography (PET) scan is an imaging method using a radioactive medium like 18F-FDG to show the metabolic activity of different tissues to reveal the metabolic or biochemical function. PET/CT, the most popular PET-imaging mode at present, combines PET and CT and can simultaneously show the pathophysiological changes and morphological structure of the lesion, offering more information for the early diagnosis and differential diagnosis of tumors. Cook, G. J. R. and his colleagues found that 18F-FDG PET uptake was higher in MPNSTs than in benign neurofibromas, and the heterogeneity was more pronounced in MPNSTs. The first-order heterogeneity parameter was discriminatory in SUVmax, which exhibited significant differences in benign and malignant lesions of neurofibromas (21) (**Figure 4**). Further studies showed that high-order features could distinguish benign from malignant tumors, but the discriminatory ability was weaker compared with the usage of SUVmax (21). One of the limitations of FDG-PET/CT is that there is a significant overlap of the SUV values between benign and malignant lesions. A prospective trial on this problem demonstrated that although the SUVmax values of benign and malignant lesions overlapped, the FDG uptake of all malignant lesions was greater than 3.15 (22). Another clinical study showed that the detection sensitivity of SUV value for asymptomatic malignant lesions was 100%, the negative predictive value was 100%, and the specificity was 45.1% (23). Consistent with those findings, another study further analyzing early and delayed imaging found similar accuracy at differentiating MPNSTs from benign NF1 but better sensitivity for delayed acquisition (24). In addition, some novel PET/CT tracers have also been used in the differentiation of benign from malignant NF1. The 68Ga-PSMA in cutaneous neurofibromas can be clearly visualized on PET/CT images and showed some differences in different lesions, suggesting the potential of 68Ga-PSMA PET/CT in the surveillance of neurofibromatosis type 1 (25). Furthermore, the addition of 11C methionine to PET/CT improved its specificity in equivocal NF1 cases (26). Among these, a study suggested amino acid preparations with half-lives and novel tracers for measuring DNA or cell membrane synthesis should also be considered (27).

However, CT imaging is not the first choice for detecting soft-tissue tumors, as MRI has higher soft-tissue resolution and provides better anatomical information than CT images in these diseases. As a result, PET/MRI, combining PET and MRI imaging, might have higher accuracy in tumor screening and diagnosis than PET/CT. Reinert, C. P. et al. analyzed FDG-PET/MRI data in patients with neurofibromatosis type 1 and found that SUV values were significantly higher in the MPNST group than in the PNF group (28). Meanwhile, there was a significant difference in the ratio of lesion SUVmean-to-liver SUVmean between MPNSTs and PNFs (28).

### Ultrasound

Ultrasound is used as an alternative regular approach to determine the peripheral neuropathic characteristics in NF1, providing valuable guidance in making a diagnosis and an operative plan (29). Beyond this, it is also considered as a potential tool for the differential diagnosis of MPNST from NF1. Peripheral neuropathies in NF1 are classified into four types by ultrasound: multiple nodular class, plexiform class, diffuse class, and mixed class (30). The multiple nodular class presents as multiple ovoids, lobulated nodes with clear boundaries, characterized by hypoechoic mixed echo in ultrasound. The plexiform class presents with thickening of long range of peripheral nerve trunks and loss of normal nerve tract structure, characterized by a pampiniform and beaded hypoechoity. The diffuse class has thickened subcutaneous tissue and fat layers, with a nodular hypoechoity. The mixed class has the diffuse class coexisting with the multiple nodular class and hypoechoic masses, often in subcutaneous muscular layers (30). When the benign neurofibroma transformed into MPNST, the ultrasound features presented as recurrence of lobulated tumors, characterized by heterogeneous echo, with plentiful blood flow signals (29). In addition, MPNST may sometimes occur in areas where peripheral neuropathies were not found previously (31).

The characteristics of benign and malignant peripheral neuropathies were further summarized manifesting in seven aspects in a study of high-resolution ultrasound: (1) The size of a benign tumor mostly was below 5 centimeters while that of MPNSTs was generally above 5 centimeters; (2) Most benign tumors grew slowly for years, while MPNSTs grew fast over weeks to months; (3) The margins of benign tumors were regular with no peritumoral edema, while the margins of MPNST were irregular and the peritumoral edema is presented; (4) Benign tumors were characterized by homogenous echo, while MPNSTs were characterized by heterogeneous echo; (5) Benign tumors were often solitary, but the MPNSTs were infiltrative; (6) Regarding to vascularization, benign tumors presented as hierarchic, and MPNSTs presented as stenotic, occlusive, trifurcated, and archaic vascular pattern; (7) In contrast-enhanced ultrasonography, no enhancement was seen in benign tumors, while peripheral enhancement with central nonenhancement presented in MPNSTs (32).

### Radiogenomics

In recent years, scientists have focused on combining imaging technology with other biological information and managed to provide imaging parameters biological explanations. Radiogenomics is a specific example of combining imaging features and genomic profiles (33). Neurofibromatosis type 1 (NF1) is an autosomal dominant disorder (1), which means that the occurrence and development of NF1 are tightly related to the gene mutation. It was reported that loss of the *CDKN2A* locus at 9p21 and mutation of the *TP53* gene might lead to the malignant transformation to MPNST. Also the loss-of-function mutations in EED and SUZ12 genes was related to MPNST, resulting in the loss of expression of trimethylated histone 3 at lysine residue 27 (H3K27me3) (34). Several studies recently have focused on the relationship between genetic phenotypes and imaging characteristics of NF1. Liu Y et al. divided the *NF1* mutations into five mutation domains (MDs) according to their biochemical functions. They also categorized the MRI features into six groups, including histogram statistics features, image gradient features, run-length (RL) texture features, gray level co-occurrence matrix texture features, shape-based features, and second-order moment features (35). Clinical characteristics were also added, and the study suggested a strong association among phenotypes, image feature patterns, and *NF1* mutation type and domains (35). Another study found that a special imaging feature of some NF1 patients on MRI, the neurofibromatosis type 1 bright objects (NBOs), was correlated with the mutation type of the *NF1* gene (36). NBOs were more likely to appear in the NF1 patients with frameshift variants than splicing or missense variants (36).

## Discussion and Future Directions

Malignant transformation of patients with NF1 can be detected by various methods, such as clinical manifestations and pathological characteristics. Three clinical symptoms, pain, enlargement of the mass, and neurological symptoms, were reported as worth evaluating (37). However, in multivariate analysis, only peripheral nerve sheath tumor enlargement remained an independent high-risk factor for malignant transformation (37). Clinical features provided primary evidence for malignant transformation in NF1 patients, but there is a great need for further evidence to confirm these associations. Under these circumstances, histopathological examination is used as the gold standard for diagnosis. Moreover, NF1 could be divided into six diagnostic categories: neurofibroma (NF), neurofibroma with atypia, cellular neurofibroma, ANNUBP, low-grade MPNST, and high-grade of MPNST according to the pathological characteristics of the tumor (34). However, tissue biopsy is an invasive process that is not suitable for every follow-up of NF1 patients during their lifetime. Therefore, reliable, noninvasive, and widely available tools are in great need.

Many studies have been devoted to the development of various image-based techniques to distinguish malignant lesions from benign NF1 tumors, including MRI, CT, PET, and ultrasound.

MRI, as a mature radiological method, has high potential in the clinical practice for differentiation of NF1 and MPNST. The “target sign” is the particular sign of benign tumors in T2-weighted imaging (11). In addition, the number of tumors, the peripheral enhancement pattern, the perilesional edema-like zone, and the presence or absence of intratumoral cystic lesions are also the key points in distinguishing benign and malignant tumors (7). However, current reported experiences-based differentiation is highly subjective, which are hard to popularize for the NF1 which is a relatively rare type of tumor. There is an urgent need for establishing objective standards for distinguishing MPNST from benign NF1. Noticing the current research highlights in radiomics and artificial intelligence-assisted diagnosis in multiple types of cancer, we propose extracting high-throughput MRI imaging features from NF1 and MPNST patients and applying deep learning methods. A machine learning model developed to automatically identify benign and malignant neurofibromas might achieve the purpose of early screening of patients with MPNST by a relatively objective and easily popularized tool.

CT is commonly used to observe bone, joint, and soft tissue lesions. Advanced computed tomography (CT), such as CT perfusion and dual-energy CT, helped distinguish multiple types of malignant tumors from benign head and neck lesions (19). However, this anatomic imaging method was proven to be ineffective to distinguish MPNST from benign NF1 (22). With the development of computer technology, the application of deep learning and artificial intelligence models has provided new possibilities for CT in the differential diagnosis of MPNST from benign NF1.

Compared to CT and MRI imaging, PET/CT combines anatomical, functional, and metabolic information of the lesion. At present, there are many clinical studies on PET/CT in differentiating benign and malignant neurofibromas based on significant differences in parameters such as SUVmax and 18F-FDG uptake (21–23). However, MRI has higher soft-tissue resolution and provides better anatomical information than CT images in these diseases, suggesting PET/MRI might have higher accuracy in tumor screening and diagnosis. Clinical studies have also reported defects of these methods: MRI had limited sensitivity for detecting MPNST, while the metabolic activity of MPNST was not always a reliable indicator of histopathologic tumor grade (38). Moreover, clinical studies of PET/MRI in differentiating benign from malignant neurofibromas are rare. But this technique is still believed to have a prosperous future in clinical usage for NF1 patients, which need more explorations in the future.

Ultrasound is a reliable, convenient, and cost-effective method for the differentiation of benign from malignant NF1, The characteristics of benign and malignant peripheral neuropathies were further summarized manifesting in seven aspects in a study of high-resolution ultrasound (32), offering a sort of differential standard for malignant transformation in individuals with NF1. Though ultrasound is not well suited for whole-body tumor volume evaluation, it is highly valuable in the diagnosis and clinical assessment of NF1 and related MPNST. Compared to PET-CT and whole-body MRI, ultrasound is radiotoxicity-free and relatively economical. In addition, ultrasound might contribute to distinguishing features of interest for investigation by MRI (29). Despite the above, ultrasound is not currently widely applied in the clinic. One reason might be the scarcity of sufficient clinical studies in this area, causing a lack of universal clinical guidelines. With the increasing clinical application of this method in the future, ultrasound might become a convenient and reliable screening method to differentiate MPNST from benign PNF.

In conclusion, multiple imaging modalities play essential roles in distinguishing MPNST from benign NF1. All these methods have their own strengths and weaknesses, such as limited sensitivity, high cost, or difficulties in whole-body assessment. More importantly, the results are not convincing enough due to the limited number of recruited patients in current studies.

Further studies are needed to solve these problems, and we recommend the following aspects be taken into consideration in future studies: (1) Combination of different imaging methods. Different imaging methods have their tendencies, and the combination could better exploit their strengths and circumvent their weaknesses. (2) Combination of computer technologies such as artificial intelligence with these imaging methods. As neurofibromatosis type 1 is a relatively rare disease, most clinical doctors, especially those in remote areas, have limited experience in reading radiological images of NF1 patients. AI models could acquire and analyze the information quickly and even exhibited better performance than doctors. The development of AI models could easily spread, which would be convenient and efficient for NF1 patients’ lifelong follow-up at their local hospital. (3) Association between radiological images and other omics. One of the directions of current studies in radiology imaging is how to explain the image parameters such as grey value differences with biological significance. In this article, we searched for the possible relationship between NF1 radiology images and genomic profiles and the results are presented. The combination of the two could probably be applied to the early discovery or even early prevention of MPNST developing from benign neurofibroma of NF1 patients. Though current studies are superficial, we believe further studies will improve our understanding of radiogenomics. In addition, the combination of radiology and histopathology is worth exploring. Although no study has completely explained the correlation of histology and radiology in NF1, a retrospective study analyzing the three-dimensional T1-weighted MR images of NF1 patients suggested that patients with NF1 had higher subcortical volumes and thicker cortices in selected regions, particularly in the hippocampus, amygdalae, cerebellar white matter, ventral diencephalon, thalamus, and occipital cortex (39). This study demonstrated the histological changes as part of the reasons for the variation on radiological images.

## Conclusion

**Figure 5:**
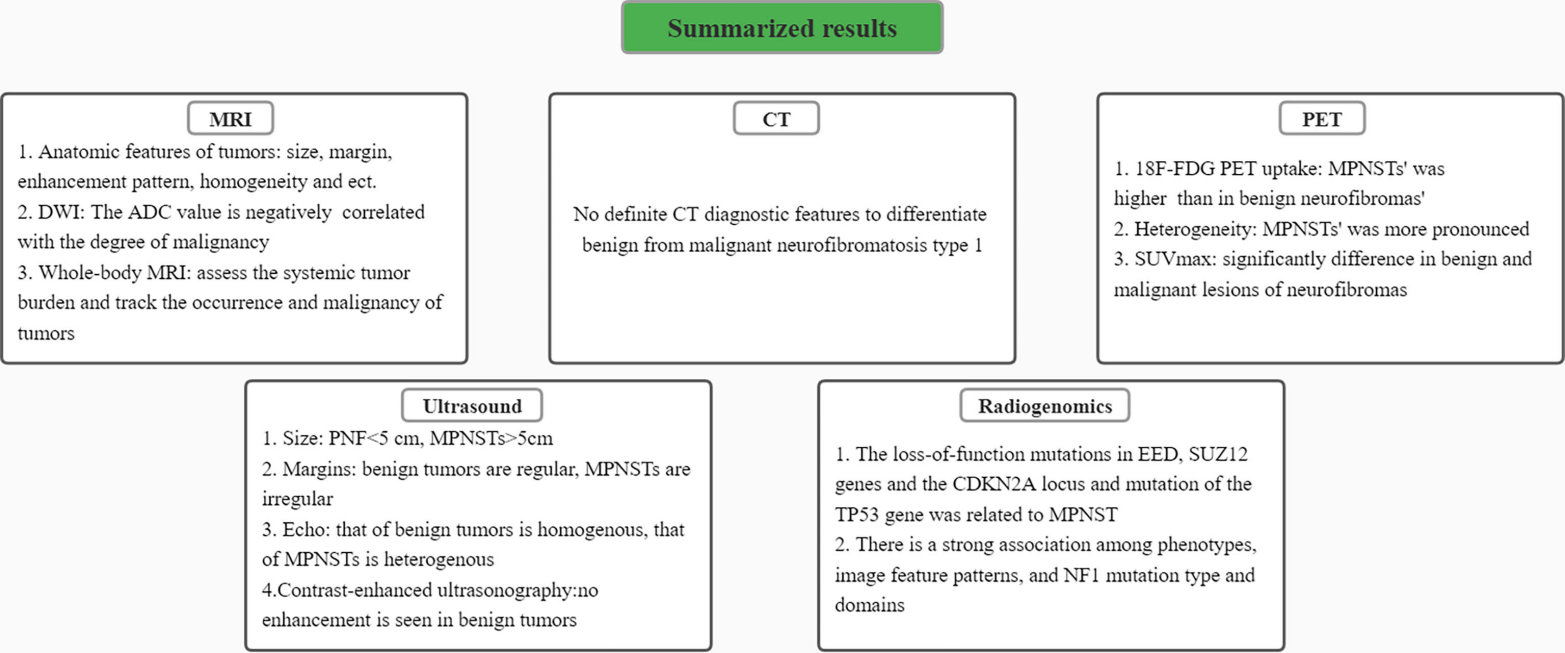
A summary diagram of results. Characteristics of differentiating benign peripheral nerve sheath tumors from malignant ones in MRI, CT, PET, ultrasound images and radiogenomics were respectively presented.

A summary diagram of image-based characteristics of differentiation of benign and malignant peripheral nerve sheath tumors in individuals with NF1 is presented (**Figure 5**). Although studies in this area are still in the early stages and mostly lack of large cohorts, current data have implicated the exciting potential roles of medical radiological imaging in the differential diagnosis of MPNST from benign NF1 at early stage and have even promoted further understanding and evaluation of this disease. With further studies in the future, we are confident in the prospect of a more significant role of these radiological imaging methods in the clinical diagnosis, follow-up, and treatment of NF1 and related tumors.

## Author Contributions

JL, J-NH, M-HW, and C-JW contributed to the conception of the study and wrote the manuscript. JL, J-NH, and M-HW contributed significantly to collection and assembly of data. W-HJ, Z-YN, C-JW, MC, and Z-CW help with the writing-review, editing and supervision. All authors read and approved the final manuscript.

## Funding

This work was supported by grants from The 15th undergraduate training program for innovation of Shanghai Jiao Tong University school of medicine (1521X311); “Chenguang Program” supported by Shanghai Education Development Foundation (SHEDF) (19CG18); Shanghai Rising Star Program supported by Science and Technology Commission of Shanghai Municipality (20QA1405600); Science and Technology Commission of Shanghai Municipality (19JC1413); Natural Science Foundation of Shanghai (22ZR1422300); Shanghai Municipal Key Clinical Specialty (shslczdzk00901); Innovative research team of high-level local universities in Shanghai (SSMU-ZDCX20180700). National Natural Science Foundation of China (82102344)

## Conflict of Interest

The authors declare that the research was conducted in the absence of any commercial or financial relationships that could be construed as a potential conflict of interest.

## Publisher’s Note

All claims expressed in this article are solely those of the authors and do not necessarily represent those of their affiliated organizations, or those of the publisher, the editors and the reviewers. Any product that may be evaluated in this article, or claim that may be made by its manufacturer, is not guaranteed or endorsed by the publisher.
